# The Investigation of Microplastics Abundance in Raw Water for Treated Water on the Primary Irrigation Canal of Karawang, West Java, Indonesia

**DOI:** 10.1155/tswj/4705541

**Published:** 2026-06-01

**Authors:** Gina Lova Sari, Ahsanal Kasasiah, Marsah Rahmawati Utami, Setyo Budi Kurniawan, Mhd. Fauzi, Nur Ridha Amethysia, Andini Siti Rohmana, Adinda Melianda Nainggolan

**Affiliations:** ^1^ Study Program of Environmental Engineering, Faculty of Engineering, Universitas Singaperbangsa Karawang, Karawang, Indonesia; ^2^ Study Program of Pharmacy, Faculty of Health Science, Universitas Singaperbangsa Karawang, Karawang, Indonesia; ^3^ Research Centre for Environmental and Clean Technologies, National Research and Innovation Agency of Indonesia (BRIN), Jakarta Pusat, Indonesia, brin.go.id

**Keywords:** Citarum Watershed, Karawang, MPs, primary irrigation canal, raw water for treated water

## Abstract

The abundance of microplastics (MPs) in the aquatic environment is increasingly threatening the sustainability of aquatic ecosystems and human health. Moreover, conventional raw water treatment for treated water used for drinking water supply has not been able to remove MPs, which can cause biomagnification and accumulation in the human body. Information regarding the distribution and characteristics of MPs, which contaminate raw water for treated water, still needs to be provided, which becomes the aim of current research. The objectives of this research are to determine the abundance and characterization of MPs in raw water for treated water in the primary irrigation canals of Karawang, which originates from the Citarum Watershed. Fifty water samples were taken along 50.00 km of primary irrigation canals with 1.00 km from each point by the volume reduction method using two modified manta trawls in duplicates. MPs were prepared, observed, and analyzed using the National Oceanic and Atmospheric Administration method, Olympus BX‐41 microscopes, and attenuated total reflectance Fourier‐transform infrared spectrophotometry (ATR‐FTIR), respectively. The results showed that MPs had contaminated raw water for treated water along the primary irrigation canals of Karawang as much as 48.33–339.17 particles/L, which was dominated by less than 1.00 mm of particles and fragments in size and shapes, respectively. The polymer of contaminated MPs was dominated by polyethylene, polypropylene, and polyvinyl chloride with similarities of 80.11%–92.26%, 81.26%–87.44%, and 88.96%. The development and improvement of management for water resources, solid waste, wastewater, and drinking water supply systems in Karawang are recommended to address the potential for MPs contamination, consumption, and biomagnification to humans.

## 1. Introduction

Microplastics (MPs), which are plastic particles smaller than 5 mm, have been recognized as one of the increasingly alarming global environmental issues [1]. MPs are found in almost all environmental compartments, including the sea, rivers, lakes, sediments, soil, and even the air [2, 3]. Their widespread presence stems from the degradation of larger plastics and their direct use in industrial and domestic products [4, 5]. International studies show that MPs can accumulate in aquatic organisms and potentially move along the food chain, ultimately endangering human health [6–8]. Some studies have even found MPs in drinking water, sea salt, and human tissue, raising serious concerns about global food safety and public health [9–11].

The problem of MPs is also related to raw water treatment systems for drinking water supply [12, 13]. Various reports reveal that conventional drinking water treatment plants in various countries have not been able to completely eliminate MPs [14–16]. This condition poses a risk of chronic exposure to humans through water consumption [17, 18]. With the increase in global plastic production and weak waste management in many countries, the risk of MPs accumulation in aquatic environments is expected to be even higher in the future [19–21]. Therefore, research on the distribution, characteristics, and impact of MPs on raw water sources is an important priority in the international environmental research agenda.

In Indonesia, the issue of MPs has become a concern, with MPs found in inland waters such as rivers and wastewater treatment plants (WWTPs) [22, 23]. Citarum Watershed is the largest and longest river in West Java, Indonesia, with an area of 661.015 ha and 297 km, respectively. It is located at 106°51 ^′^36 ^″^‐107°51 ^′^ East Longitude and 7°19 ^′^‐6°24 ^′^ South Latitude and has been divided into upstream, middle, and downstream zones. In these zones, the Citarum Watershed is used as a source of raw water for drinking purposes, agricultural irrigation, fisheries, and hydroelectric power generation [24]. Nonetheless, the Citarum Watershed has been reported as one of the most plastic‐contaminated rivers in Indonesia due to an inadequate and unmanaged plastic waste disposal system [25–29]. Plastic waste in the Citarum Watershed gradually breaks down into MPs, ranging from 0.10 *μ*m up to 5.00 mm. These MPs, composed of harmful hydrocarbon compounds, can leach toxic chemicals and adsorb persistent organic compounds, posing severe environmental and health issues [30]. The water contamination with MPs is further exacerbated by the inadequate wastewater treatment system, both in household and industrial activities in the area [31, 32].

Several previous studies have reported facts about the presence of MPs in the Citarum Watershed, where as many as 5.85 ± 3.28 particles/L [25], 0.71 × 104–4.59 × x10^5^ particles/km^2^ [32]; and 60.00 ± 30.00 particles/L [31] were found in the Ciwalengke River (upstream zone), Jatiluhur Reservoir (middle zone), and Muara Gembong River (downstream zone), respectively. It shows that MPs have high stability and may transfer to other areas flowed by MPs‐contaminated water [29, 30]. It threatens the quality of freshwater along the Citarum Watershed, primarily used as raw water for treated water used for drinking purposes, because it can be one of the main routes for MPs‐biomagnification, accumulating in the human body, ending posing a significant health risk [33]. As reported by recent studies, MPs have been found in human feces [34, 35] and human placenta [36], potentially leading to impaired body function and cancer due to carcinogenic and persistent [33, 37].

The Citarum River Basin flows to Karawang, located in the downstream zone, with water from the Walahar Dam channeled to the Muara Gembong River and the main irrigation canal for approximately 50 km to Telukambulu Village. This canal is used by the community for domestic needs and is a source of raw water for three drinking water companies, making it potentially contaminated with MPs. However, information on the presence of MPs in raw water is still limited, even though this data is important to support sustainable water treatment strategies and technologies. Therefore, this study is aimed at identifying the abundance and distribution of MPs in the raw water of the main Karawang irrigation canal and at raising public awareness of its impact so that comprehensive preventive measures can be implemented.

## 2. Materials and Methods

### 2.1. Water Sample Collection

**Figure 1:**
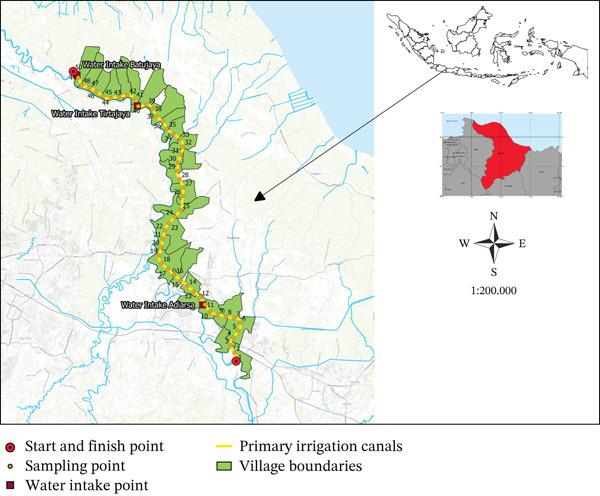
Map of MPs sampling point along the primary irrigation canal of Karawang.

Water samples were collected from 50 locations along the primary irrigation canal of Karawang, with intervals of 1.00 km between each sampling point (Figure 1), using rafting boats passing through 33 villages in Karawang. Water sampling every 1.00 km along the main irrigation channel was chosen to ensure even data distribution so that water quality conditions from upstream to downstream could be represented, while facilitating the identification of significant change points due to domestic, agricultural, or industrial waste inputs. This interval was also considered dense enough to detect variations in water quality while remaining efficient in terms of energy, time, and cost. These villages are grouped into nine subdistricts: Klari, Majalaya, East Karawang, West Karawang, Rengasdengklok, Kutawaluya, Jayakerja, Tirtajaya, and Batujaya, which are densely populated areas.

Furthermore, water sampling was performed by volume‐reduce method using two modified manta trawl nets [38, 39] with pore sizes of 500 and 333 *μ*m, equipped with a 600‐mL collection bottle as the cod end. Manta trawl nets are towed on both sides of the boat at a depth of 0.00–0.40 m with a suitable distance to avoid wave and boat propeller interferences at a speed of 2.00–3.00 knots [32, 34, 38, 40] as far as 1.00 km to collect water samples. Afterward, the nets and cod end were rinsed using distilled water to ensure no MPs were left behind [34, 41]. Each sample was taken in duplicates, composited, mixed thoroughly, and then transferred into a sterile glass bottle and stored at 4°C [42].

### 2.2. Sample Preparation

Prior to analysis, each composited water sample was prepared using the National Oceanic and Atmospheric Administration method to remove organic matter and separate MPs from the water [34, 42]. Water samples were filtered using Whatman GF/C filter with a diameter of 120 mm and pores of 1.2 *μ*m, with the aid of a vacuum pump under a pressure of 0.02 MPa, and then transferred to a beaker. Furthermore, 20.00 mL of 0.05 M Fe (II) and 20.00 mL of H_2_O_2_ were added sequentially to separate metals and remove organic matter from MPs. The samples were mixed and homogenized using a magnetic stirrer for 30 min at 60.00°C–75.00°C. Then, 6.00 g of NaCl was added to every 20.00 mL of homogenized sample to increase the density to facilitate the separation of organic matter and MPs using a density separator.

### 2.3. Analysis and Characterization of MPs

Analysis was performed to identify the size, shape, and color of MPs using an Olympus BX‐41 microscope with 10 times magnification. The MPs found were counted manually and classified into different shapes (fiber, fragment, film, and foam) and sizes (smaller than 1.00 mm, 1.00 to less than 3.00 mm, and 3.00 to less than 5.00 mm). The abundance of MPs was determined by comparing the number of MPs found with the volume of filtered water samples [34, 42].

The abundance of MPs=the amount of MPs particlevolume of filtered water L 

Furthermore, the MPs polymer was analyzed using attenuated total reflectance Fourier‐transform infrared spectrophotometry [43], known as ATR‐FTIR (Thermo Scientific Nicolet iS10), with a wavelength of 4000–650 cm^−1^.

## 3. Results and Discussions

### 3.1. The Abundance and Characteristics of MPs in the Primary Irrigation Canal of Karawang

**Figure 2:**
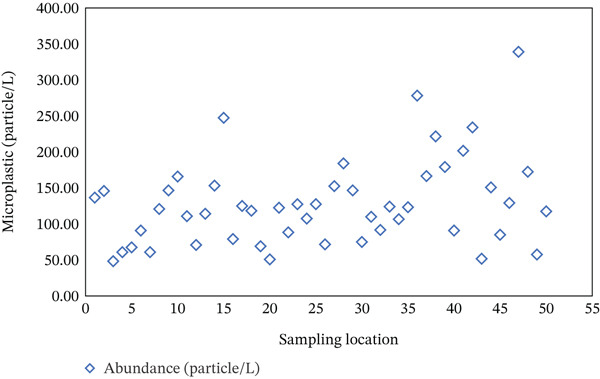
Abundance of microplastics.

**Figure 3:**
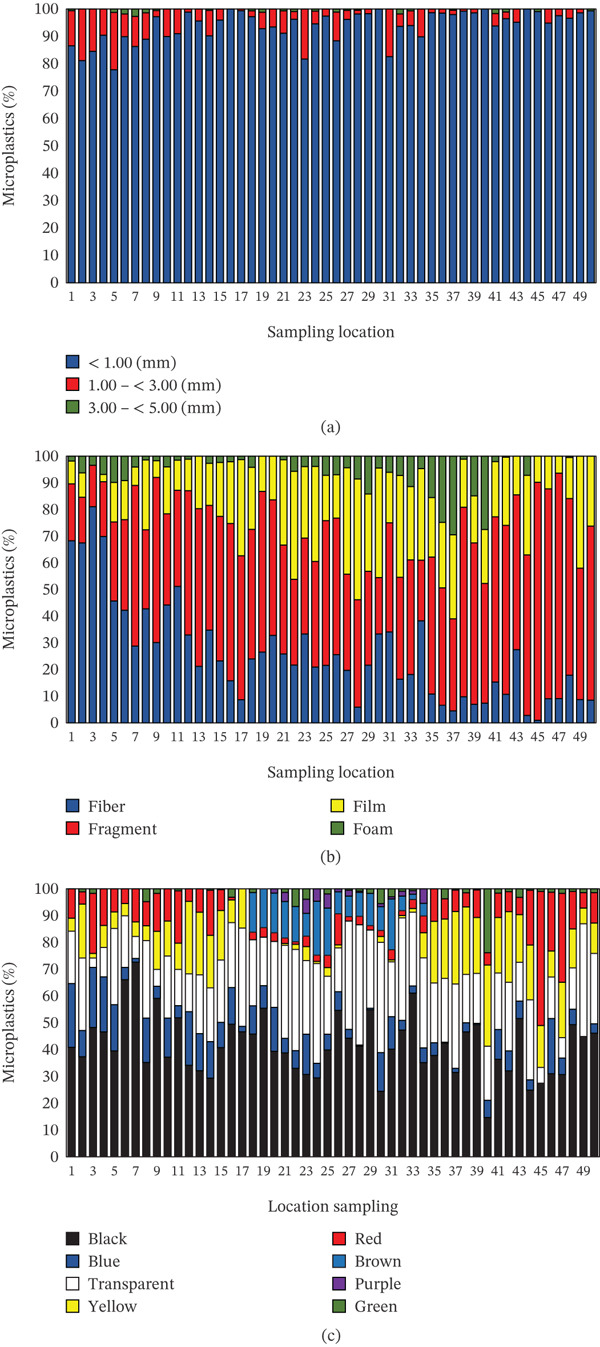
(a) Size, (b) shape, and (c) color of microplastics.

MPs were found in all raw water for treated water used for drinking purposes along the upstream irrigation canals in Karawang with a range of 48.33–339.17 particles/L and dominated by fragments measuring less than 1.00 mm, reaching an average of 63.80 particles/L (Figures 2 and 3). Different findings were found in other West Java waters that are sources of raw water for identical utilization, where MPs were found in the Ciwalengke River [25], Cimandiri River [44], Jatiluhur Reservoir [32], and Muara Gembong River [31, 41] at 5.85 ± 3.28 particles/L, 6.85–74.00 × ×10^5^ particles/L, 0.07–4.59 × 10^5^ particles/km^2^, and 38.00–86.00 particles/L, respectively. Based on current and previous research, it is known that fragment, film, and fiber in the size of 1.00–5.00 mm dominated the MPs contamination in West Java waters.

**Figure 4:**
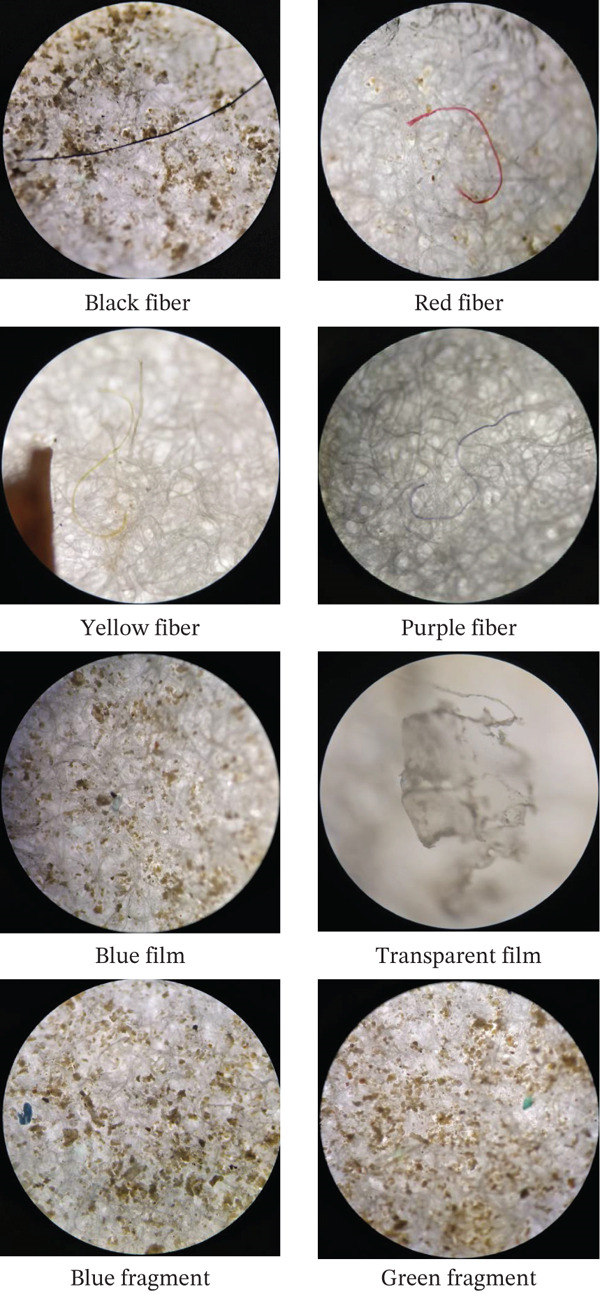
Shapes and colors of MPs from a microscope (10 times magnification).

The results of the study indicate that MPs at the observation site varied in size, shape, and color (Figure 3). The shape and color of MPs observed using a microscope can be seen in Figure 4. In terms of size (Figure 3a), the fraction < 1 mm was the most dominant compared with other categories (1–3 mm and 3–5 mm). This indicates that most of the plastic has been mechanically and chemically degraded in the water [45–47]. Ecologically, these small sizes are dangerous because they are more easily suspended in the water column and ingested by various organisms, ranging from zooplankton to food fish [1, 48]. Thus, the dominance of particles < 1 mm reflects the high ecological risk as well as the potential for MPs accumulation in the food chain.

From the aspect of shape (Figure 3b), the data show that fiber is the most commonly found MPs morphology, followed by fragments, film, and foam. Fibers mostly originate from synthetic fibers in clothing, which are released during the washing process and eventually carried by wastewater flows into water bodies. In addition, fibers can also be released from fishing equipment such as nets, ropes, or nylon thread. These findings are consistent with many previous studies reporting that fibers are the most dominant form of MPs in rivers and oceans, especially in areas with high domestic and fishing activity [49, 50]. The fragments and films found most likely originate from the degradation of single‐use plastics, such as shopping bags, food packaging, or mineral water bottles, which are fragmented due to exposure to sunlight and mechanical processes [51]. Meanwhile, foam usually comes from polystyrene products, such as fast food containers, floats, or packaging materials [52, 53]. The dominance of fibers confirms that the sources of MPs pollution at the study site are closely related to household activities and the use of synthetic materials in daily activities and fishing.

Color analysis (Figure 3c) shows that black, blue, and transparent MPs dominate, whereas yellow, red, green, and purple are found in relatively smaller amounts. Black MPs are generally associated with vehicle tire abrasion, plastic combustion residues, or dark‐colored textiles [54]. Blue MPs most likely originate from fishing nets, plastic ropes, or colored synthetic fibers, which are widely used in fishing activities. Meanwhile, transparent MPs are strongly suspected to be the result of the degradation of plastic bags, water bottles, and disposable packaging that are widely used by the community [55]. Other colors found in small quantities, such as red, yellow, or purple, can be linked to specific plastic products, such as toys, household items, or colored food packaging. These color differences are important to analyze because they can provide clues about the possible sources of pollution, as well as determine the extent to which MPs have the potential to attract the attention of aquatic organisms, which often mistake brightly colored particles for natural food.tan

Overall, the distribution pattern of MPs′ size, shape, and color indicates that the sources of pollution at the research site are multisectoral. Domestic waste is the main contributor through the release of fibers from clothing and synthetic products, whereas the fishing sector contributes blue fiber‐shaped particles, and single‐use plastics contribute transparent film and foam. This condition illustrates the complexity of MPs pollution in water, where various human activities contribute simultaneously. In addition, the dominance of sizes < 1 mm indicates that plastic pollutants entering water bodies do not only come from new plastics, but also from the degradation of old plastics that are difficult to decompose, thereby cumulatively increasing the number of small particles that are more dangerous.

**Figure 5:**
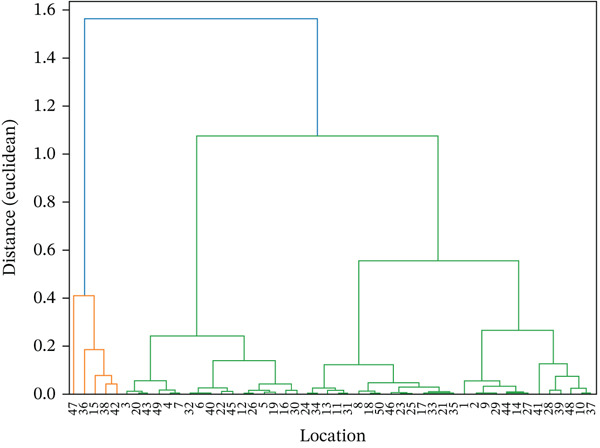
Dendogram clustering of MPs abundance.

Based on the statistical test results, the concentration of MPs at 50 sampling points along a 50‐km stretch of the river was not normally distributed (Shapiro–Wilk, *p* < 0.05), so the analysis was continued using nonparametric methods. The Kruskal–Wallis test results showed a *p* value of 0.473 (> 0.05), which means that there was no significant difference in MP concentrations between sampling points. The dendrogram clustering of MPs′ abundance can be seen in Figure 5.

The insignificant differences between sampling points indicate that the source of MP pollution in rivers does not originate from a specific point (point‐source pollution), but rather from nonpoint sources or diffuse sources, such as domestic waste, household activities, agriculture, and plastic degradation from activities along the river. The relatively even distribution may also be caused by river hydrodynamic processes, such as currents, turbulence, and sedimentation–resuspension, which play a role in spreading MPs to various locations. This condition is in line with several previous studies that reported that rivers with sufficiently long flows tend to show a homogeneous distribution pattern of MPs, especially when there are pollution sources in many locations along the riverbanks [56–58].

Although not statistically significant, small variations between sampling points may still occur due to local factors, such as population density, direct waste disposal into rivers, and industrial activities around the waterways. However, the Kruskal–Wallis test results show that these variations are not strong enough to produce statistically significant differences. Ecologically, this finding is important because it shows that MPs have become a widespread pollutant along the river, meaning that pollution control efforts cannot be focused on a single source point but must instead be carried out through a comprehensive management strategy across the entire watershed.

The permutational multivariate analysis of variance test results show that MP characteristics along the river flow differ significantly when analyzed based on size, shape, and color. In terms of size, the distribution of MPs was divided into three categories, namely < 1 mm, 1.00–< 3.00 mm, and 3.00–< 5.00 mm, with a pseudo‐*F* value of 2.431 and *p* = 0.019. A *p* value of less than 0.05 indicates that the differences in size distribution between sampling points are statistically significant. This indicates that the plastic degradation process does not occur uniformly along the river. In some segments, MPs measuring < 1 mm are more dominant, usually formed as a result of further fragmentation of larger plastics [59–61]. Meanwhile, sizes of 1.00–< 3.00 mm and 3.00–< 5.00 mm are more likely to originate from plastics that entered the environment in the form of larger particles, such as packaging pieces, bottles, or single‐use plastics [55]. These size variations may reflect differences in current intensity, human activity, and microenvironmental conditions in each river segment.

The difference is more pronounced in terms of MPs′ shape, with a pseudo‐*F* value of 5.284 and *p* = 0.001. This difference indicates that the composition of MP shapes differs significantly between sampling locations. The four main shapes found are fibers, fragments, films, and foam. Fibers are generally associated with textile waste, clothing fibers released during washing, and the degradation of fishing nets. Fragments usually originate from hard plastic debris, either from household appliances or degraded construction materials. Films are often found in plastic bags or food wrappers, whereas foam is closely related to the use of Styrofoam for food packaging and goods transportation. The fact that the distribution of different forms varies significantly between sampling points reinforces the assumption that the sources of plastic pollution in this river are highly diverse and depend on the dominant activity in a particular segment. For example, densely populated residential areas are more likely to contribute fibers from domestic waste, whereas commercial areas or economic activities along the riverbanks may contribute more fragments and films.

In addition to size and shape, the color of MPs also showed significant differences between sampling points, with a pseudo‐*F* value of 2.426 and *p* = 0.010. The colors found included black, blue, transparent, yellow, red, brown, purple, and green. This color variation can be an important clue to the origin and entry route of plastics into rivers. For example, blue and black are often associated with textile fibers, tires, or fishing nets, whereas transparent colors generally originate from single‐use plastics such as bags or mineral water bottles. Red, yellow, and green usually come from colored packaging or household plastic products, whereas brown and purple can be associated with specific types of plastics from household or industrial equipment. Differences in color distribution between sampling points indicate that, in addition to variations in sources, the degradation process also plays a role. Transparent plastics tend to undergo photodegradation more easily, whereas dark‐colored plastics are usually more resistant to sunlight, resulting in different distributions in nature.

**Figure 6:**
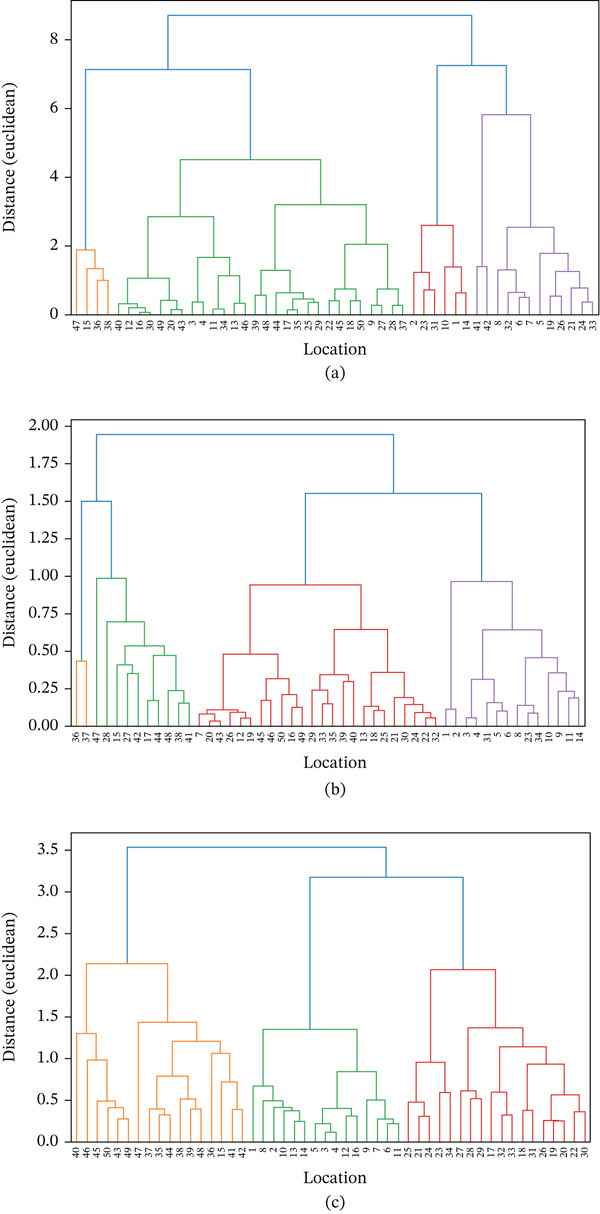
Dendrogram clustering of (a) MP size, (b) shape, and (c) color.

Overall, these results confirm that although the total concentration of MPs found along the 50‐km river flow is relatively homogeneous (based on the previous Kruskal–Wallis results), the characteristics of MPs such as size, shape, and color show significant variation. The dendrogram clustering based on the size, shape, and color of MPs is presented in Figure 6a, Figure 6b, and Figure 6c. In other words, MPs are evenly distributed, but the dominant properties and types of MPs differ between river segments. This is closely related to differences in human activities and land use along the riverbanks. The upstream segment may receive more input from domestic activities, the middle segment from trade or small industrial activities, and the downstream segment from the accumulation of various sources of pollutants carried by the current.

From an ecological perspective, the variation in size, shape, and color of MPs indicates diverse sources of pollution, ranging from households and fisheries to industry. Therefore, control measures should not only focus on reducing the total amount of MPs but also target specific sources, such as fibers from domestic waste and films and foams from single‐use plastics. Rivers serve as the main transport route for MPs to the sea, so mitigation must be carried out holistically at the watershed level, taking into account the varying characteristics and sources of pollution in each segment [61].

**Figure 7:**
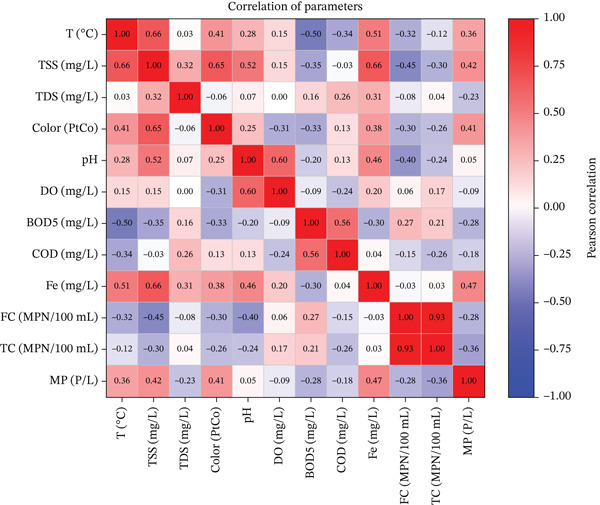
Correlation of physical, chemical, and biological parameters with MP.

The results of Pearson′s analysis (Figure 7) show a close relationship between MPs and physical–chemical parameters, particularly TSS, color, temperature, and Fe metal. The values of these physical, chemical, and biological parameters are derived from this published research [62]. A moderate positive correlation between MP and TSS (*r* = 0.42) and color (*r* = 0.41) indicates that high suspended particles and turbidity play an important role in carrying or maintaining the presence of MP in the water column. This is reasonable because MPs are small and light, making them easily associated with suspended sediments, especially in river segments with dense human activity such as domestic waste disposal and commercial activities. Meanwhile, a positive correlation with temperature (*r* = 0.36) indicates that an increase in temperature can accelerate the process of plastic degradation into microfragments. In addition, the strong correlation between MP and Fe (*r* = 0.47) suggests links to industrial or urban pollution, where both MPs and heavy metals may be released together and share similar distribution patterns.

On the other hand, the correlation between MP and organic water quality parameters such as BOD_5_ (*r* = −0.28) and COD (*r* = −0.18) shows a relatively weak negative relationship. This indicates that the presence of MPs is not directly related to organic load, as MPs tend to be more associated with inorganic pollutants or nonbiodegradable solids. Meanwhile, the negative relationship with DO (*r* = −0.09) is also weak, indicating that dissolved oxygen levels in rivers are not directly affected by MP concentrations. A similar pattern is seen in the correlations with biological parameters such as fecal coliform (FC) (*r* = −0.28) and total coliform (TC) (*r* = −0.36), which are relatively small. These findings suggest that MPs′ presence is driven more by physical–chemical factors than by microbiological contamination from domestic and agricultural waste.

Ecologically, these results confirm that MPs in river waters have a unique distribution pattern, influenced by physical processes (currents, sedimentation, and suspension) and inorganic pollutant sources, whereas their relationship with organic and biological pollutants is relatively insignificant. Therefore, MP pollution control strategies should not be limited to organic waste treatment or sanitation, but should also target the control of suspended solids, the management of single‐use plastic waste, and the monitoring of industrial waste that has the potential to release heavy metals and plastics into the environment.

The dominant MPs found using ATR‐FTIR analysis were polyethylene (PE), polypropylene (PP), and polyvinyl chloride (PVC) (Table 1), reflecting the widespread use of these materials in packaging, textiles, and household products. Their presence in Karawang′s irrigation canals highlights poor waste management and shows that plastic pollution also threatens freshwater infrastructure essential for agriculture. With PE and PP remaining dissolved and PVC accumulating in sediments, these thermoplastics pose ecological and health risks while emphasizing the urgency of better plastic waste control.

**Table 1:** The dominant polymer of MPs along the primary irrigation canal of Karawang.

The shape of MPs	The type of polymer	The percentage of similarity	Peak
Fiber	Polyethylene	87.93%–88.59%	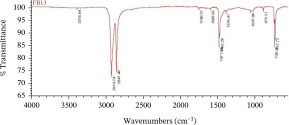
	Polypropylene	84.45%–87.44%	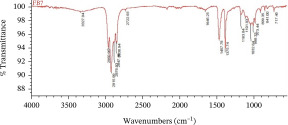
Film	Polyethylene	80.69%–92.26%	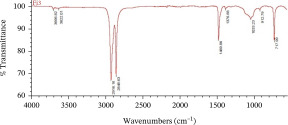
	Polyvinyl chloride	88.96%	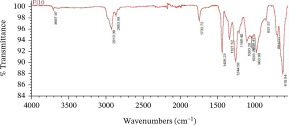
Fragment	Polypropylene	81.26%–85.21%	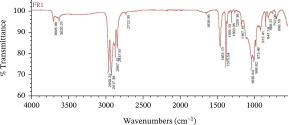
	Polyethylene	80.11%–89.31%	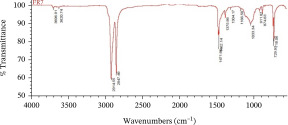
Foam	Bentonite (brown)	66.56%	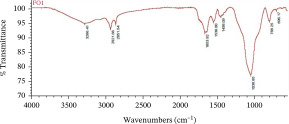

### 3.2. Analysis of Abundance of MPs in Raw Water for Treated Water Along the Primary Irrigation Canal of Karawang

In Central Java and its surroundings, raw water for drinking purposes reported to be contaminated with MPs are the Bengawan Solo River and Winongo River, which have lower abundances than Karawang. MPs contamination was found in the Bengawan Solo River at 0.05–78.67 particles/L, dominated by fiber [63–65]. Meanwhile, in the Winongo River, MPs were found at 7.90–11.95 particles/L, dominated by fragments and films [66].

MP contamination in raw water sources for drinking purposes in East Java, particularly in the Surabaya River, is as many as 3450.00–63,380.00 particles/L and is dominated by films [39, 67]. Another MP contamination was found in the Banyuurip River at 7780.00 particles/L and is dominated by fragments and fibers [68]. Pratama et al. [69] added that the flow of the Surabaya River in Bambe and Tawang Sari each contained MPs, as many as 440,000 and 320,000 particles/L, which were dominated by fibers. Fitriyah et al. [70] also reported that 49.00–95.00 particles/L of MPs were detected in the Kalimas River, also dominated by fibers.

Moreover, MP contamination is not limited to Java. It has also been found in non‐Java waters. In the Babura and Sikambing rivers, raw water sources for drinking purposes in Medan City, MPs were found at levels of 70.00–170.00 particles/L, with the most common forms being films and fragments [71]. The Mahakam River, a raw water source for drinking purposes in East Kalimantan, was also found to be contaminated by MPs, with as many as 13.00–21.00 particles/L, predominantly in fibers [72].

Figure 2 presents a high abundance of MPs detected in the upstream of primary irrigation canals at Points1 and 2, with levels as high as 136.67 and 145.83 particles/L, respectively. These points, located at the beginning of the water transfer from the Citarum Watershed to the Karawang irrigation canals, increase the possibility of MPs from the previous zone being carried away by the current upstream of the Jatiluhur Reservoir, as reported by Ramadan and Sembiring [41]. Although filters have been provided in the area, their performance is less than 100.00% [73], where they can only remove macroplastics, not MPs.

**Figure 8:**
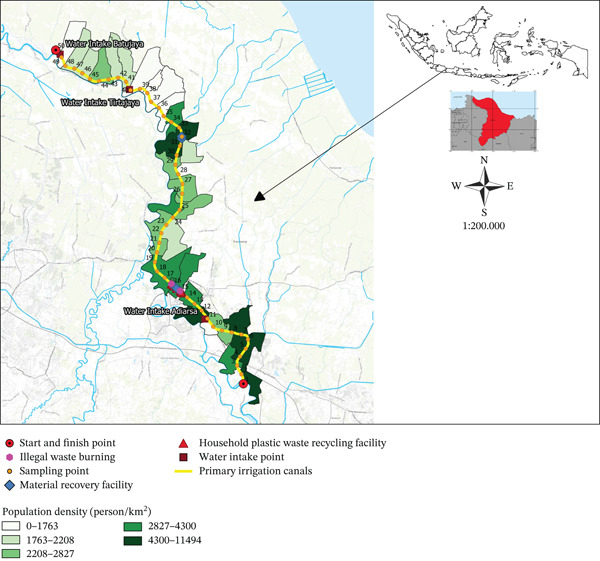
Sources of microplastics.

Moreover, it is essential to understand that MP contamination from Point 3 to Point 50 is primarily influenced by the domestic and commercial activities of the local populace people residing along the canals, a pattern observed in Central Java, East Java, non‐Java, and other countries falling under the primary source category [74, 75]. These activities, particularly those related to personal care, such as bathing and washing, directly utilize water in the primary irrigation canals of Karawang and introduce microbeads into it (Figure 8). These microbeads, commonly found in products like toothpaste and soap, have an average size of 250.00 *μ*m [76], and their use can release around 4000.00 particles [73, 77]. Previous studies in Flanders, Belgium, have found that each person contributes an estimated 1450 MPs particles/L through domestic wastewater discharge every day [78].

Meanwhile, the community‐owned laundry businesses dominate commercial activities along the primary irrigation canal of Karawang. Their wastewater is directly discharged into the canals without any prior treatment. Notably, the potential release of fibers from continuous laundry activities, estimated at up to 411,000 kg/year, is a significant concern for water quality [73, 79, 80]. It is consistent with the findings of the current study that fiber is dominant MPs‐contaminated. The release of fibers from laundry activities is intricately linked to the characteristics of the textile/fabric, detergent, and washing mechanism used [80]. Each of these factors plays a crucial role in the amount and type of fibers released. This activity also has an impact on increasing the concentration of oil and grease, BOD, and COD along the Karawang primary irrigation canals which exceed the quality standards for raw water for treated water and drinking purposes according to Government Regulation of the Republic of Indonesia No. 22 of 2021 [81].

Microbeads and fibers released from domestic wastewater [73, 82] could end up in the primary irrigation canals of Karawang, then contaminating and increasing the abundance of MP to the raw water for treated water there. The presence of dominant fibers in the water at the primary irrigation canals of Karawang and other areas can be attributed to various materials, such as ropes from fish farming and fishing activities. This includes fishing rods, nets, and aquaculture equipment [79, 83].

Another commercial activity that could be a source of MPs release is the household plastic waste recycling facility (H‐PWRF). Based on the observations, it is known that there are several H‐PWRFs along the primary irrigation canal of Karawang. The activity of H‐PWRF consisted of easy washing, chopping, and sun‐drying (Figure 8), which is not equipped with a WWTP. It might drive wastewater from the washing process to directly infiltrate the soil or flow into primary irrigation canals [73, 75].

Several studies have reported that untreated H‐PWRF wastewater can contain MPs ranging from 27.00 particles/L to 1.12 × 1011 particles/L, which is one of the primary sources of MP release [84–87], either overflowing by gravitation or runoff into surface water [73]. Furthermore, sun‐drying is done by exposing chopped plastic to direct sunlight during the day. This allows the particles to be carried into the primary irrigation canals through air deposition due to their tiny particles [73, 75]. Meanwhile, MPs may still be released through the wastewater produced by MRFs that have WWTP. [86] reported 21.00 particles of MP/L in treated wastewater dominated by fragments. Furthermore, Umarie and Bagastyo [87] also reported that wastewater and sludge processed from WWTPS of PRF contained 98.00 particles of MPs/L and 364.81 particles of MPs/kg, respectively, which were dominated by fragments. Fragments dominated the MPs released from the H‐PRF, as Suzuki et al. [85] found that 98.00%–99.00% of MP from the facilities were fragments. This finding reinforced the condition that fragments are one of the dominant MPs found in raw water for drinking purposes in the primary irrigation canal of Karawang. This finding was ironic, as the leading role of PRF is to reduce plastic waste through recycling, but the process can still increase the abundance of MPs in the environment.

The air deposition of MPs to surface water, a process that can occur as a result of open waste burning [75], is a pressing issue. MPs, with a density ranging from 900.00 to 1330.00 kg/m^3^ [88], are released into the air and subsequently deposited into primary irrigation canals. The volatile organic compounds released alongside MPs during open burning can significantly increase the ecotoxicity of these waters up to 7.44% [75]. Moreover, the impact of human activities on water systems is particularly evident in the case of tire wear due to abrasion with asphalt. As pointed out by Enfrin et al. [89] and Vercauteren et al. [78], the release of MPs from tire wear and their subsequent transport into surface water via runoff has become a new issue, with an estimated annual release of approximately 246.00 t.

In addition, the presence of fragments and films in the primary irrigation canal of Karawang can be linked to the photodegradation and thermo‐oxidation of plastic waste in the water [26]. This issue arises from mismanaged waste practices [75, 90, 91]. Numerous inadequate public waste storage facilities, including illegal dumping sites and open burning of waste, have been identified along the primary irrigation canal, as illustrated in Figure 8. These factors contribute to the uncontrolled spread of MPs. It is noteworthy that the unmanaged plastic waste surrounding the primary irrigation canals in Karawang predominantly consists of thermoplastics, such as plastic bottles and bags. These materials either degrade naturally or are burned by the community [75], resulting in the production of fragments and films.

Current research indicates that various community activities contribute to the contamination of raw water for treated water used for drinking purposes in Karawang. Key factors include direct water usage, the discharge of domestic wastewater, wastewater from the H‐PWRF, transportation, and the improper disposal of plastic waste. Analysis of the primary irrigation canals reveals the presence of MPs in the following quantities: 3190.00 particles/L (49.70% fragments), 1449.00 particles/L (22.58% fibers), 1395.00 particles/L (21.73% films), and 384.17 particles/L (5.99% foams).

The significant finding of MP contamination in the raw water for treated water used for drinking purposes poses a serious threat to human health that might be correlated with the low implementation of clean and healthy living behaviors. It is due to inadequate wastewater management and drinking water supply systems in Karawang, which might have affected the number of diseases in Karawang such as diarrhea [33] as many as 20,472 cases in 2023 [92].

On the other hand, raw water in primary irrigation canals is also used as a source of water for agriculture in Karawang, which potentially increases the contamination of MPs in rice fields then translocated to rice [73]. This underscores the urgent need for research and coordinated immediate action to address the issue of MP contamination in the water systems through the improvement of water management, wastewater management, and drinking water supply systems.

## 4. Conclusion

This research has unequivocally confirmed that MPs raw water for treated water used for drinking purposes along the primary irrigation canal of Karawang is currently contaminated by MPs. This contamination, a direct result of the improper management of solid waste, wastewater, and water resources, is an urgent issue that demands immediate attention. The total abundance of MP in the primary irrigation canals ranged from 48.33 to 339.17 particles/L for fibers, fragments, films, and foams with particle sizes less than 1.00 mm, 1.00–3.00 mm, and 3.00 to less than 5.00 mm. The results showed that MPs in the size and shape of less than 1.00 mm and fragment, respectively, were dominant in the current study. Meanwhile, the contaminated‐MP polymers were detected as PE, PP, PVC, and bentonite (brown) with similarities of 80.11%–92.26%, 81.26%–87.44%, 88.96%, and 55.56%. Furthermore, the direct use of raw water by communities along primary irrigation canals to meet household and agricultural needs can have serious health implications. Enhancing water resource management, wastewater management, and drinking water supply systems are essential components of a coordinated effort that is the responsibility of all stakeholders.

## Funding

This research was supported by the Ministry of Education, Culture, Research and Technology of the Republic of Indonesia under Grant Number 244/E5/PG.02.00PT/2022.

## Conflicts of Interest

The authors declare no conflicts of interest.

## Data Availability

The data that support the findings of this study are available from the corresponding author upon reasonable request.
